# Investigation of intra-day variability of gaseous measurements in sheep using portable accumulation chambers

**DOI:** 10.1093/jas/skab132

**Published:** 2021-05-03

**Authors:** Edel O’ Connor, Nóirín McHugh, Tommy M Boland, Eoin Dunne, Fiona M McGovern

**Affiliations:** 1 Teagasc, Animal and Grassland Research and Innovation Centre, Athenry, Co. Galway, Ireland; 2 School of Agriculture and Food Science, University College Dublin, Belfield, Dublin 4, Ireland

**Keywords:** intra-day variability, methane, portable accumulation chamber, sheep

## Abstract

Portable accumulation chambers (PAC) enable short-term spot measurements of gaseous emissions including methane (CH_4_), carbon dioxide (CO_2_), and oxygen (O_2_) consumption from small ruminants. To date the differences in morning and evening gaseous measurements in the PAC have not been investigated. The objectives of this study were to investigate: 1) the optimal measurement time in the PAC, 2) the appropriate method of accounting for the animal’s size when calculating the animal’s gaseous output, and 3) the intra-day variability of gaseous measurements. A total of 12 ewe lambs (c. 10 to 11 months of age) were randomly selected each day from a cohort of 48 animals over nine consecutive days. Methane emissions from the 12 lambs were measured in 12 PAC during two measurement runs daily, AM (8 to 10 h) and PM (14 to 16 h). Animals were removed from Perennial ryegrass silage for at least 1 h prior to measurements in the PAC and animals were assigned randomly to each of the 12 chambers. Methane (ppm) concentration, O_2_ and CO_2_ percentage were measured at 5 time points (T1 = 0.0 min, T2 = 12.5 min, T3 = 25.0 min, T4 = 37.5 min, and T5 = 50.0 min from entry of the first animal into the first chamber) using an Eagle 2 monitor. The correlation between time points T5-T1 (i.e., 50 min minus 0 min after entry of the animal to the chamber) and T4-T1 was 0.95, 0.92, and 0.77 for CH_4,_ O_2_, and CO_2,_ respectively (*P* < 0.01). The correlation between CH_4_ and CO_2_ output and O_2_ consumption, calculated with live-weight and with body volume was 0.99 (*P* < 0.001). The correlation between the PAC measurement recorded on the same animal in the AM and PM measurement runs was 0.73. Factors associated with CH_4_ production included: day and time of measurement, the live-weight of the animal and the hourly relative humidity. Results from this study suggest that the optimal time for measuring an animal’s gaseous output in the PAC is 50 min, that live-weight should be used in the calculation of gaseous output from an animal and that the measurement of an animal’s gaseous emissions in either the AM or PM does not impact on the ranking of animals when gaseous emissions are measured using the feeding and measurement protocol outlined in the present study.

## Introduction

The agricultural sector globally is estimated to account for 23% of anthropogenic greenhouse gas emissions (GHG; Arneth et al., 2019); therefore, a reduction in the GHG emissions from this sector is required to ensure that global average temperature does not increase above 2 °C above pre-industrial times under the Paris agreement (UN, 2015). Methane (CH_4_) is a potent GHG and has a greater global warming potential (100-yr global warming potential of 28) in comparison to carbon dioxide (CO_2_; 100-yr global warming potential of 1; Allen et al., 2018). Total agricultural CH_4_ emissions globally account for 38.62% of total CH_4_ emissions (FAO, 2020).

Respiration chambers are considered the “gold standard” in the estimation of gaseous emissions from ruminants (Robinson et al., 2014). However, the respiration methodology is labor intensive, expensive, have a low animal throughput, do not measure animals in their natural environment (Bhatta et al., 2007). Respiration chambers may also reduce feed intake and alter feeding behavior, which may cause an underestimation of daily CH_4_ emissions compared with what would occur in the animal’s actual production system (Bickell et al., 2014; Jonker et al., 2014). Therefore, short-term spot sampling methods are required on farms that enable the measurement of animals in their natural grazing environment. Such short-term spot sampling methods include laser CH_4_ detectors (Sorg et al., 2018), the Greenfeed system (Rapid City, South Dakota; C-Lock Inc.; Manafiazer et al., 2016; Hailemariam et al., 2020; Manafiazer et al., 2020a), and Portable accumulation chambers (PAC; Jonker et al., 2018). Portable accumulation chambers were used in the current study as they are suitable for measuring gaseous emissions from small ruminants in grazing systems (Jonker et al., 2018) and allow for measurements from a larger number of animals in a shorter period of time compared with other methods. The PAC allow for a 1 h measurement period of accumulated gas (Jonker et al., 2018) and there has been research into reducing the measurement time where the animals are in the PAC (Goopy et al., 2011; Robinson et al., 2015), but more investigation may be necessary. Additionally, it is necessary to account for the size of the animal when measuring emissions using PAC and current studies have assumed that the animal’s live-weight is acceptable (Goopy et al., 2016; Robinson et al., 2016; Jonker et al., 2018), rather than calculating the actual volume of the animal. Finally, despite there being a known diurnal pattern of gaseous emissions (Jonker et al., 2014; Hammond et al., 2015; Manafiazar et al., 2020a), has not been investigated into the differences in an animals ranking from PAC estimates measured in the morning compared with the afternoon. Therefore, the objectives of this study were to: 1) investigate the length of time animals need to be in the PAC to obtain an accurate gaseous measurement; 2) evaluate the suitable variable to account for animal size when calculating gaseous output; and 3) examine the intra-day variability of gaseous emissions from sheep.

## Materials and Methods

Data were generated from an experiment undertaken on nulliparous Texel and Suffolk ewe lambs (c. 10 to 11 months of age) in late winter and early spring 2020 at the Teagasc Animal and Grassland Research and Innovation Centre, Athenry, Co. Galway. The study was approved by the Teagasc Animal Ethics Committee (TAEC0496-2020) and the Health protection regulatory authority (AE19132/P098).

### Portable accumulation chambers

**Figure 1.**
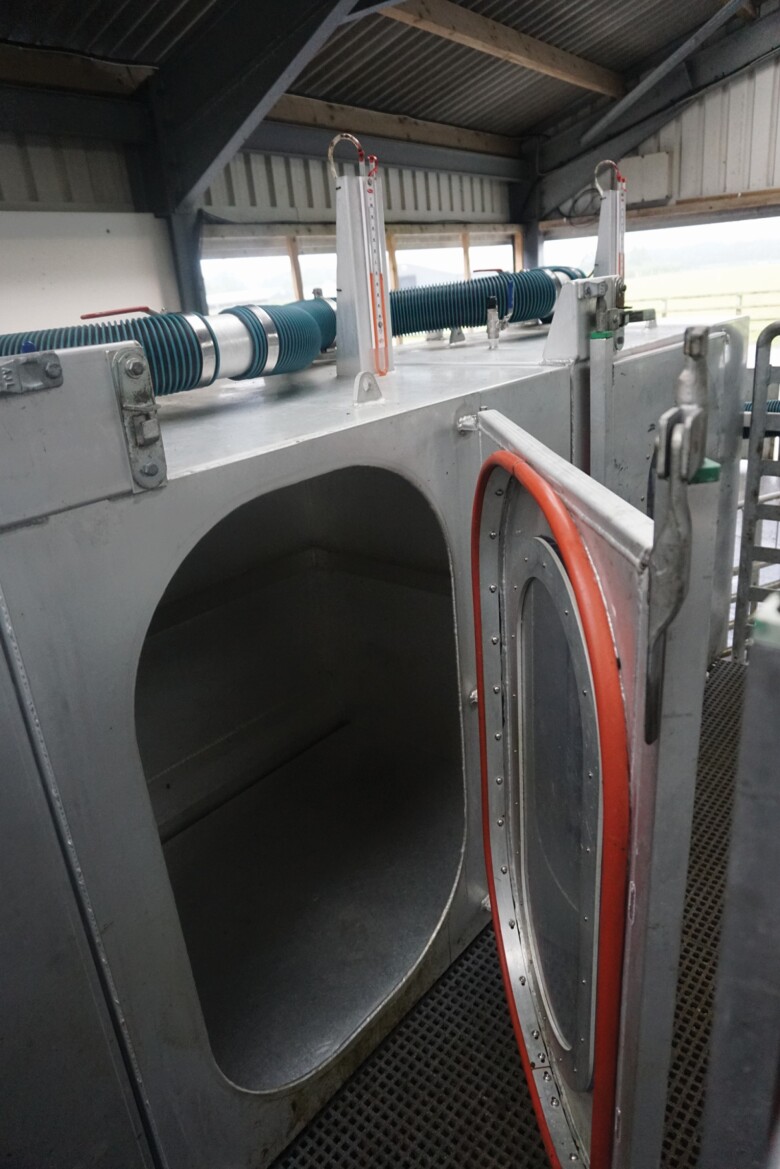
Portable accumulation chamber.

For the purpose of this experiment CH_4_, oxygen (O_2_), and CO_2_ measurements were obtained using 12 PAC, as described by Jonker et al. (2018). Briefly, the PAC are rectangular shaped compartments composed of polycarbonate sheets, which are 1.17 m length × 1.15 m height × 0.615 m width, with an internal volume of 827 liters (Figure 1). The chambers are air tight and are fitted with manometers to monitor pressure and leaks within each chamber. A sampling valve on top of each chamber allows for the monitoring of gas measurements while animals are placed in the chambers. In this study, gases were measured using an RKI Eagle 2 monitor (Weatherall Equipment and Instruments Ltd, UK) whereby the probe of the monitor was inserted into the sampling valve and a stable reading was recorded. After completion of the gaseous measurements the sampling valve was closed immediately. To ensure the accuracy of gas measurements, daily gas checks (prior and post each PAC measurement run) of the Eagle 2 monitor were conducted using standard calibration gases of 100 ppm, 1,000 ppm, and 5,000 ppm CH_4_.

A gas extraction vacuum system is fitted on each of the 12 chambers which allowed for the removal of all residual gases from the chambers at the end of each measurement run. For the purpose of this experiment, gas measurements were taken on each chamber prior to entry of the animal and in all incidences read 0 ppm CH_4_, 20.9% O_2_, and 0% CO_2_. Upon entry of the animal into the individual chamber, the door was closed and the measurement run commenced. In this experiment, gas measurements for CH_4_ (ppm), O_2_ (%), and CO_2_ (%) were taken at five specific time points at and after entry of the animal into the chamber (T1 = 0.0 min (animal entry), T2 = 12.5 min, T3 = 25.0 min, T4 = 37.5 min, and T5 = 50.0 min) using the RKI Eagle 2 monitor (Weatherall Equipment and Instruments Ltd, UK). The exact time of each measurement was also recorded. Ambient temperature, atmospheric pressure, and relative humidity were also recorded separately for each measurement run.

### Skeletal measurements

Skeletal measurements were obtained for all lambs prior the commencement of the experiment, whereby a tape was used to measure both abdominal girth and body length (in centimeters) of each animal. Abdominal girth was measured as the circumference at the widest part of the abdomen. Body length was measured as the length from the top of the head midway between the ears to the tail head of each lamb. These measurements were subsequently used to calculate the body volume (BV dm^3^) of each lamb using the formula derived by Paputungan et al. (2015):


$$\begin{array}{l} BV=\frac{\left[BL\times \pi {\left(\frac{\left(\frac{AG}{2}\right)}{\pi }\right)}^{2}\right]}{1000} \end{array}$$ (1)

where BL is the body length of the animal (cm), π is 3.14, and AG is the abdominal girth of the animal (cm).

### Data collection

The experiment was conducted over nine consecutive days in late winter and early spring 2020. On each of the nine experimental days, 12 ewe lambs were randomly selected from a cohort of 48 ewe lambs and each animal was randomly assigned to 1 of the 12 individual PAC. The experiment was conducted over two measurement runs, in the morning (between 8 to 10 h; referred to hereafter as AM) and evening (between 14 to 16 h; referred to hereafter as PM); across each measurement run the same 12 animals were chosen for the AM and PM measurements each day. Forty-five lambs were measured on average 2.4 times over the study. All ewe lambs were housed indoors for the duration of the experiment and were offered a diet of Perennial ryegrass silage *ad libitum*. One hour prior to the commencement of measurements the 12 selected animals were removed from feed (Robinson et al., 2015) and weighed using a Prattley weighing scales (O’ Donovan Engineering Co. Ltd, Cork, Ireland). Upon completion of the AM measurements the animals were returned to their penning area where Perennial ryegrass silage was offered *ad libitum* for at least 2 h prior to the commencement of the PM measurements.

The gaseous measurements of CH_4_ obtained for each animal over each measurement run were converted to liter/hour (l/hour) using the equation:


$$\begin{array}{l} CH_{4}\left(1/hour\right)=\left(\frac{\left(\frac{Methane_{x}\text{-}Methane_{y}}{Time_{x}\text{-}Time_{y}}\;\times \;60\right)\;\times \;\left(827\text{-}\left(live\text{-}weight\right)\right)}{1,000,000}\right) \end{array}$$ (2)

where CH_4_ (l/hour) is the CH_4_ emissions quantified in liters per hour, Methane_x_ is methane output in ppm at time point x, Methane_y_ is CH_4_ output in ppm at time point y, Time_x_ is the time at time point x, Time_Y_ is the time at time point y, and live-weight is the live-weight of the animal in kg.

A similar equation was used to convert the O_2_ and CO_2_ measurements obtained for each animal over each measurement period


$$\begin{array}{l} Gas\left(1/hour\right)=\left(\frac{\left(\frac{Gas_{x}\text{-}Gas_{y}}{Time_{x}\text{-}Time_{y}}\;\times \;60\right)\;\times \;\left(827\text{-}\left(live\text{-}weight\right)\right)}{100}\right) \end{array}$$ (3)

where gas (l/hour) is O_2_ or CO_2_ quantified in liters per hour, Gas_x_ the percentage O_2_ or CO_2_ at time point x, Gas_y_ is the percentage O_2_ or CO_2_ at time point y, Time_x_ is the time at time point x, Time_y_ is the time at time point y and live-weight is the live-weight of the animal in kg.

For each of the above equations, the final gas volume obtained in l/hour can be extrapolated up to a g/day value using an equation similar to the ideal gas law as described by Jonker et al. (2018) whereby


$$\begin{array}{ll} CH_{4}\left(g/day\right)=\;C & H_{4}\left(1/hour\right)\;\times \;\frac{\left(Press\;\times \;0.1\right)}{\left(8.3145\;\times \;\left(Temp+273.15\right)\right)}\;\\ & \times \;16\;\times \;1440 \end{array}$$ (4)

where CH_4_ (l/hour) is CH_4_ emissions quantified in liters per hour, press is the pressure expressed in hectopascals and temp is the temperature expressed in degrees Celsius, 16 is the molecular weight of CH_4_, and 1,440 is the number of minutes in the day. This equation was also used for the calculation of CO_2_ and O_2_; however, the molecular weight was changed from 16 in the case of methane to 44 for CO_2_ and 32 for O_2_.

The respiratory quotient (RQ) was calculated as the number of moles of CO_2_ produced divided by the number of moles of O_2_ consumed. Total daily gas production (mol/day) was calculated as the daily moles of CH_4_ produced plus the daily moles of CO_2_ produced.

Outliers were removed ± 3 standard deviations from the mean gas volume produced (l/hour) to give 108 individual animal records with 216 observations from 45 animals.

### Chemical analysis

Representative samples of the silage offered were collected daily. Samples were dried at 60 °C for 48 h using a Memmert ‘Excellent’ forced air circulation oven (Memmert GMBH, Schwabach, Germany) to determine dry matter (DM) content, which on average was 22.66 ± 1.36% DM. Samples were bulked based on day of measurement and were subsequently analyzed for DM, ash, neutral detergent fiber (Van Soest, 1963), acid detergent fiber, crude protein (Leco FP-428; Leco Australia Pty Ltd, Baulkham Hills, New South Wales, Australia).

### Statistical analyses

To investigate the stability of a gaseous measurement over each time point (i.e., T1 to T5 for each lamb), Lin’s concordance correlation coefficients were calculated between each time point (SAS Inst. Inc., Cary, NC). Similarly the regression coefficients between each time point were calculated in a fixed effects model using PROC GLM.

To quantify the within-day variability (i.e., AM vs. PM measurements) in gaseous emissions, a mixed model which accounted for repeated records was developed using PROC MIXED, using the following model:


$$\begin{array}{l} Y_{ijk}=\mu +T_{i}+A_{j}+D_{k}+e_{ijk} \end{array}$$

where Y_*ijkl*_ is the dependent variable of gaseous production in l/hour (i.e., CH_4_, O_2_, and CO_2_), µ is the population mean, T_*i*_ is the random effect of time of measurement (*i* = AM or PM), A is the random animal effect (*j* = 48), D is the repeated effect of measurement date (*k* = 9), and e_*ijk*_ is the residual effect. In addition a homogeneity test of AM and PM variances was investigated using a fixed effects model in PROC GLM.

To investigate the suitability of using live-weight or body volume to calculate gaseous emissions, the correlation between gaseous measurements and live-weight or body volume were calculated using PROC CORR. Factors associated with gaseous emissions were determined using linear mixed models in PROC MIXED, using the following model:


$$\begin{array}{ll} Y_{ijklmnopq\;}= & \;\mu +T_{i}+BRD_{j}+D_{k}+LW_{l}+C_{m}\\ & +P_{n}+Temp_{o}+H_{p}+e_{ijklmnopq} \end{array}$$

where Y_*ijklmnopq*_ is the dependent variable of gaseous production in l/hour (i.e., CH_4_, O_2_, and CO_2_), µ is the population mean, T_*i*_ is the effect of time of measurement (*i* = AM or PM), BRD_*j*_ is the effect of breed (*j* = 2), D is the effect of measurement date (*k* = 9), LW_*l*_ is the effect of live weight (*l* = 38 to 56), C_*m*_ is the effect of chamber number (*m* = 1 to 12), P_*n*_ is the effect of pressure (*n* = 991 to 1040), Temp_*o*_ is the effect of temperature (*o* = 2 to 14), H_*p*_ is the effect of humidity (*p* = 54 to 91), and e_*ijklmnopq*_ is the residual effect. A multiple regression model was built up using stepwise forward–backward regression; the significance threshold for entry and exit of variables into/from the model was set at 1%.

## Results

The average live-weight of the animals was 45.8 ± 4.3 kg. The mean abdominal girth and body length were 101.08 ± 5.83 cm and 89.51 ± 4.59 cm, respectively. Among all animals body volume averaged 73.13 ± 9.84 dm^3^. The average temperature, pressure, and humidity across the 9-d period was 9.30 ± 2.34 °C, 1007.59 ± 15.26 hPa, and 76.7 ±8.53%, respectively.

The mean output of CH_4_ measured across the 50 min was 0.0088 ± 0.0029 l/hour. A similar value was observed at the time point T4-T1 (i.e., 37.5 min minus 0 min after entry of the animal to the chamber) of 0.009 l/hour; however, the range of mean CH_4_ output varied from 0.0081 (T5-T4) to 0.0096 l/hour (T2-T1) across all time points investigated. These values can be extrapolated using equation (4) to provide a grams per day value of 8.62 g/day for T5-T1 and a value of 8.87 g/day for T4-T1. The mean O_2_ consumed over the 50 min was 0.34 ± 0.14 l/hour and ranged from 0.21 l/hour (T2-T1) to 0.43 l/hour (T3-T2) across all time measurements. The mean CO_2_ produced across the 50 min was 0.21 ± 0.07 l/hour with the same mean carbon dioxide produced at T4-T1 (±0.08). The mean CO_2_ produced ranged from 0.18 (T4-T3) to 0.21 (T2-T1) l/hour across the time points. These values can be extrapolated up using equation (4) to a g/day value of 561.45 g/day (T5-T1), a lower value can be seen at T4-T1 of 526.39 g/day. The average RQ was 0.66 (SE 0.01) with a mean total gas production of 13.30 mol/day.

### Relationships between measurement time-points

The correlation between CH_4_ output measured across all five time points is shown in Table 1 and ranged from 0.07 (T4-T3 and T3-T2; i.e., 37.5 min minus 25 min and 25 min minus 12.5 min after entry of the animal to the chamber; *P* = 0.31) to 0.95 (T5-T1 and T4-T1; *P* < 0.01). The corresponding regression coefficient observed between time points T5-T1 and T4-T1 was 1.02 (SE 0.02) and a *R*^2^ of 0.92 (Table 1). For the O_2_ consumed, the correlation between time points is shown in Table 2 and ranged from -0.08 (T4-T3 and T3-T2; *P*=0.24) to 0.94 (T5-T1 and T4-T1; *P*<0.01). The regression coefficient values ranged from 0.06 (SE 0.09; T3-T2 and T4-T3) to 1.41 (SE 0.08; T5-T1 and T2-T1) with *R*^2^ ranging from 0.00 (T4-T3 and T3-T2) to 0.90 (T5-T1 and T4-T1). The correlations observed between time points for CO_2_ shown in Table 3 ranged from -0.30 (T4-T3 and T3-T2; *P*<0.01) to 0.86 (T5-T1 to T4-T1; *P*<0.01). The corresponding regression coefficient and *R*^2^, between time points T5-T1 and T4-T1 for CO_2_ was 0.88 (SE 0.04) and 0.74, respectively. As T5-T1 and T4-T1 had regressions coefficients closest to 1 and the greatest *R*^2^, the remainder of the paper will focus on the gas measurements calculated from time points T5-T1 and T4-T1.

**Table 1.** The regression coefficients (standard error in parentheses; above the diagonal) and correlations (below the diagonal) between methane measured across various time points measured over a 50-min period in the portable accumulation chambers^1,2^

Time Points	T5-T1	T4-T1	T3-T1	T2-T1	T5-T2	T4-T2	T3-T2	T5-T3	T4-T3	T5-T4
T5-T1		1.02 (0.02)	1.02 (0.04)	1.03 (0.07)	0.99 (0.03)	1.02 (0.04	1.00 (0.07)	0.94 (0.04)	0.95 (0.08)	0.92 (0.06)
T4-T1	0.95*		0.98 (0.04)	0.99 (0.06)	0.87 (0.03)	0.99 (0.04)	0.94 (0.06)	0.79 (0.05)	0.99 (0.07)	0.59 (0.07)
T3-T1	0.82*	0.85*		1.01 (0.04)	0.77 (0.06)	0.63 (0.05)	0.98 (0.04)	0.51 (0.07)	0.41 (0.08)	0.51 (0.06)
T2-T1	0.66*	0.71*	0.85*		0.6 (0.08)	0.46 (0.07)	0.47 (0.06)	0.53 (0.08)	0.27 (0.06)	0.44 (0.07)
T5-T2	0.92*	0.85*	0.63*	0.40*		1.05 (0.03)	0.93 (0.07)	0.97 (0.03)	1.04 (0.07)	0.89 (0.06)
T4-T2	0.83*	0.87*	0.60*	0.36*	0.92*		0.82 (0.06)	0.82 (0.04)	1.05 (0.05)	0.46 (0.07)
T3-T2	0.49*	0.51*	0.69*	0.38*	0.51*	0.53*		0.50 (0.08)	0.25 (0.06)	0.46 (0.07)
T5-T3	0.80*	0.71*	0.36*	0.31*	0.89*	0.79*	0.21*		1.14 (0.06)	0.79 (0.06)
T4-T3	0.52*	0.58*	0.16	0.16	0.62*	0.75*	0.07	0.79*		0.22 (0.08)
T5-T4	0.67*	0.46*	0.45*	0.36*	0.71*	0.41*	0.33*	0.70*	0.22*	

^1^Time points: T5 = 50 min, T4 = 37.5 min, T3 = 25 min, T2 = 12.5 min, and T1 = 0 min.

^2^The color gradient indicates that the closer the value is to 1 the darker the gray color is while the closer the value is to 0 the lighter the color is.

*Correlation differed (*P <* 0.01) from zero.

**Table 2.** The regression coefficients (standard error in parentheses; above the diagonal) and correlations (below the diagonal) between oxygen measured across various time points measured over a 50-min period in the portable accumulation chambers^1,2^

Time Points	T5-T1	T4-T1	T3-T1	T2-T1	T5-T2	T4-T2	T3-T2	T5-T3	T4-T3	T5-T4
**T5-T1**		1.05 (0.02)	1.29 (0.05)	1.41 (0.08)	0.85 (0.03)	0.88 (0.05)	1.14 (0.09)	0.68 (0.05)	0.60 (0.08)	0.71 (0.06)
**T4-T1**	0.94*		1.20 (0.04)	1.23 (0.07)	1.00 (0.05)	0.84 (0.04)	1.08 (0.07)	0.48 (0.05)	0.56 (0.07)	0.42 (0.06)
**T3-T1**	0.81*	0.88*		1.03 (0.04)	1.10 (0.08)	0.89 (0.07)	0.93 (0.05)	0.41 (0.10)	0.11 (0.06)	0.28 (0.05)
**T2-T1**	0.55*	0.61*	0.76*		0.82 (0.12)	0.55 (0.10)	0.40 (0.07)	0.58 (0.12)	0.19 (0.09)	0.56 (0.10)
**T5-T2**	0.84*	0.76*	0.54*	0.26*		1.09 (0.04)	1.29 (0.09)	0.84 (0.05)	0.82 (0.08)	0.78 (0.06)
**T4-T2**	0.70*	0.77*	0.53*	0.20*	0.86*		1.19 (0.07)	0.65 (0.06)	0.77 (0.06)	0.31 (0.07)
**T3-T2**	0.43*	0.50*	0.63*	0.23*	0.43*	0.56*		0.20 (0.12)	0.06 (0.09)	0.33 (0.10)
**T5-T3**	0.67*	0.52*	0.19*	0.22*	0.79*	0.59*	-0.01		1.05 (0.06)	0.84 (0.06)
**T4-T3**	0.38*	0.44*	0.01	0.07	0.48*	0.60*	-0.08	0.73*		0.22 (0.08)
**T5-T4**	0.58*	0.94*	0.79*	0.56*	0.85*	0.70*	0.40*	0.68*	0.37*	

^1^Time points: T5 = 50 min, T4 = 37.5 min, T3 = 25 min, T2 = 12.5 min, and T1= 0 min.

^2^The color gradient indicates that the closer the value is to 1 the darker the gray color is while the closer the value is to 0 the lighter the color is.

*Correlation differed (*P <* 0.01) from zero.

**Table 3.** The regression coefficients (standard error in parentheses; above the diagonal) and correlations (below the diagonal) between carbon dioxide measured across various time points measured over a 50-min period in the portable accumulation chambers^1,2^

Time Points	T5-T1	T4-T1	T3-T1	T2-T1	T5-T2	T4-T2	T3-T2	T5-T3	T4-T3	T5-T4
**T5-T1**		0.88 (0.04)	1.00 (0.06)	1.28 (0.10)	0.88 (0.04)	0.64 (0.07)	0.59 (0.11)	1.00 (0.06)	0.73 (0.13)	1.17 (0.11)
**T4-T1**	0.86*		0.93 (0.06)	1.19 (0.10)	0.67 (0.05)	0.86 (0.06)	0.57 (0.1)	0.72 (0.07)	1.09 (0.12)	0.35 (0.12)
**T3-T1**	0.71*	0.70*		1.09 (0.07)	0.68 (0.07)	0.34 (0.07)	0.75 (0.07)	0.28 (0.07)	-0.13 (0.11)	0.52 (0.09)
**T2-T1**	0.46*	0.46*	0.64*		0.43 (0.12)	-0.20 (0.11)	0.07 (0.08)	0.39 (0.10)	-0.20 (0.06)	0.35 (0.07)
**T5-T2**	0.82*	0.66*	0.41*	0.13		0.85 (0.06)	0.85 (0.10)	1.06 (0.05)	0.88 (0.12)	1.11 (0.10)
**T4-T2**	0.46*	0.66*	0.26*	-0.06	0.68*		0.70 (0.08)	0.48 (0.06)	1.25 (0.08)	-0.07 (0.11)
**T3-T2**	0.22*	0.26*	0.51*	-0.09	0.32*	0.42*		0.00 (0.09)	-0.14 (0.06)	0.08 (0.06)
**T5-T3**	0.69*	0.48*	0.11	0.23*	0.77*	0.42*	-0.16		0.98 (0.08)	0.92 (0.08)
**T4-T3**	0.21*	0.36*	-0.16	-0.01	0.32*	0.55*	-0.30*	0.59*		-0.14 (0.07)
**T5-T4**	0.49*	0.84*	0.68*	0.48*	0.85*	0.46*	0.20*	0.72*	0.24*	

^1^Time points: T5 = 50 min, T4 = 37.5 min, T3 = 25 min, T2 = 12.5 min, and T1 = 0 min.

^2^The color gradient indicates that the closer the value is to 1 the darker the gray color is while the closer the value is to 0 the lighter the color is.

*Correlation differed (*P <* 0.01) from zero.

### Variables used to calculate gaseous output of an animal

The correlation between BV and live-weight ranged from 0.29 (day 7) to 0.79 (day 1). When the live-weight of the animals was averaged over the 9-d experimental measurement phase, the correlation declined to 0.63 (*P* < 0.01). The mean CH_4_ output calculated using BV was 0.0085 ± 0.0028 l/hour (T5-T1; i.e., 50 min minus 0 min after entry of the animal to the chamber) and 0.0087 ± 0.0030 l/hour (T4-T1). Similar results were observed when live-weight, instead of BV, was used to calculate gaseous output, with a CH_4_ output of 0.0088 ± 0.0029 l/hour (T5-T1) and 0.0090 ± 0.0032 l/hour (T4-T1) calculated. A close to unity correlation (*r* = 0.99; *P*<0.01) was found between the CH_4_ output calculated using BV or using live-weight at both T5-T1 and T4-T1; the corresponding *R*^2^ was 0.34 and 0.31, respectively. The average O_2_ consumed calculated using BV was 0.33 ± 0.13 l/hour (T5-T1) and 0.32 ± 0.15 l/hour (T4-T1). Similar values were observed when live-weight was used in the calculation, 0.34 ± 0.014 l/hour (T5-T1) and 0.33 ± 0.16 l/hour (T4-T1). A *R*^2^ of 0.48 (T5-T1) and 0.46 (T4-T1) were found between the O_2_ consumed when calculated using BV and using live-weight, with a correlation of 0.99 (*P* < 0.01) between both time points. The mean CO_2_ produced calculated using BV was 0.20 ± 0.07 l/hour for T5-T1 and 0.20 ± 0.07 l/hour for T4-T1, while similar values were calculated using live-weight, 0.21 ± 0.07 l/hour (T5-T1) and 0.21 ± 0.08 l/hour (T4-T1). A strong correlation of 0.99 (*P* < 0.01) was observed between both time points for CO_2_ with an *R*^2^ of 0.49 and 0.38 for T5-T1 and T4-T1, respectively.

### Intra-day variability of gaseous measurements

The results of the test for homogeneity of variance indicates that there is no significant difference between AM and PM measurements for methane output derived at both T5-T1 (*P* = 0.41; Coefficient of variation (CV) = 31.99; i.e., 50 min minus 0 min after entry of the animal to the chamber) and T4-T1 (*P* = 0.14; CV = 33.28). The mean CH_4_ produced at T5-T1 in the AM measurement was 0.0078 l/hour (SE = 0.0003) while 0.0097 l/hour (SE = 0.0004) was produced during the PM measurement run. Similar results were seen at time point T4-T1 with 0.008 l/hour produced in the AM measurement run and 0.01 l/hour produced in the PM measurement run. The correlation between AM and PM measurement runs for T5-T1 and T4-T1 indicates that the values obtained for CH_4_ were precise, regardless of being measured in the AM or PM measurement runs, with a correlation of 0.73 (*P* < 0.01; SE 0.0002) for T5-T1 and 0.72 (*P* < 0.01; SE 0.0002) for T4-T1. The correlations between the AM and PM CH_4_ measurements across each day ranged from 0.51 (day 3; *P* = 0.09) to 0.90 (day 7; *P* < 0.01) using time point T5-T1, for T4-T1 the corresponding correlations ranged from 0.44 (day 1; *P* = 0.15) to 0.93 (day 2; *P* < 0.01).

The homogeneity of variance test results for O_2_ showed that 0.34 l/hour of O_2_ was consumed in the AM measurement and 0.35 l/hour in the PM measurement for time point T5-T1 while 0.32 l/hour and 0.35 l/hour were consumed in the AM and PM measurements, respectively, for T4-T1. The test indicated that there was no significant difference between AM and PM measurements for both time point T5-T1 (*P* = 0.04; CV = 40.83) and T4-T1 (*P* = 0.02; CV = 47.11). The correlations between AM and PM O_2_ consumption measurements were strong for both T5-T1 (*r* = 0.80; *P* = 0.32) and T4-T1 (*r* = 0.72; *P* = 0.01). The correlation between AM and PM O_2_ consumption measurements across each experimental day ranged from 0.51 (day 5; *P* = 0.09) to 0.94 (day 4; *P* < 0.01) for T5-T1 and between 0.38 (day 6; *P* = 0.23) to 0.94 (day 4; *P* < 0.01) for T4-T1. The homogeneity of variance test for CO_2_ at time points T5-T1 and T4-T1 indicates that there was no significant difference between AM and PM measurements (*P* ≥ 0.07) with CV of 35.94 and 37.45, respectively. The results also showed that 0.21 l/hour was produced in both the AM and PM measurements for both time points T5-T1 and T4-T1. The correlations for CO_2_ at the time point T5-T1 showed that the AM and PM measurements had a strong relationship with a correlation of 0.75 (*P* = 0.65; SE 0.005); however, at the time point T4-T1 a moderate correlation of 0.55 (*P* = 0.40; SE 0.006) was observed. The relationship within day for the AM and PM measurements of CO_2_ showed weak to strong correlations ranging from 0.19 (day 2; *P* = 0.57) to 0.73 (day 9; *P* = 0.01) using time point T5-T1. When using time point T4-T1, the correlations ranged from -0.13 (day 7; *P* = 0.68) to 0.69 (day 3; *P* = 0.01).

### Factors affecting the methane output

The factors associated with CH_4_ production (l/hour; Table 4) included: date of measurement (*P* < 0.01), time of measurement run (AM/PM; *P* < 0.01), animal live-weight (*P* < 0.01), and the relative humidity (*P* = 0.01). Methane production ranged from 0.006 l/hour (day 5) to 0.011 (day 9), while CH_4_ production was larger in the PM measurement runs compared with the AM measurement runs. For every 1 kg increase in animal live-weight and 1% increase in relative humidity, CH_4_ production increased by 0.00021 l/hour (SE = 0.00006) and 0.00006 l/hour (SE = 0.00002), respectively. Methane output did not differ based on breed of the animal, chamber number, temperature, or atmospheric pressure (*P* > 0.01).

**Table 4.** Regression coefficient (b; standard error (SE) in parenthesis) and the associated *P*-value of each factor associated with methane production (l/hour) calculated using animal live-weight (Live-weight)

Factor	Level	b (SE)	*P*-value
Day	1	0.010 (0.0005)	<0.001
	2	0.009 (0.0005)	
	3	0.008 (0.0005)	
	4	0.008 (0.0005)	
	5	0.006 (0.0005)	
	6	0.009 (0.0005)	
	7	0.009 (0.0005)	
	8	0.010 (0.0005)	
	9	0.011 (0.0005)	
Time^1^	AM	0.008 (0.0003)	<0.001
	PM	0.010 (0.0003)	
Weight		0.0002 (0.0001)	<0.001
Humidity		0.00006 (0.00002)	<0.001

^1^Where AM refers to the morning (8 to 11 h) and PM refers to evening (14 to 16 h) measurement runs.

## Discussion

Ruminant animals are a leading contributor of CH_4_ emissions (Jiao et al., 2014) and, therefore, there is a need to develop robust methodology that can allow for the measurements of CH_4_ from enteric fermentation. The main objectives of this paper were to determine the length of time in which sheep need to be placed in the PAC to achieve a ranking estimate, to determine the most appropriate variable to account for the size of the animal when calculating gaseous output and how an animal’s gaseous output varies across the day.

Although respiration chambers are considered the gold standard method of obtaining accurate gaseous emissions in ruminants (Manafiazar et al., 2020b), they are an expensive and slow method for measuring CH_4_ emissions and is not financially viable when measurements on large number of animals are required. In contrast, the PAC are a suitable low-cost, rapid method of measuring CH_4_ (Jonker et al., 2018). High genetic correlations have been reported between respiration chamber and PAC measurements ranging from 0.62 to 0.67 for CH_4_ production (Jonker et al., 2018). While the PAC does not reflect absolute values of CH_4_ production, it allows for the identification of high and low emitting sheep, thus ranking the animals (Goopy et al., 2011; Jonker et al., 2018) and the objective of this study was to establish if the animals rank consistently throughout the day on their gaseous emissions (i.e., from morning to evening). As the PAC is a spot sampling method, animals are removed from feed approximately 1 h prior to measuring to avoid capturing the post-feeding spike in CH_4_ production which has been shown to occur 45 to 140 min post feeding (Crompton et al., 2011). This study does not take into account all possible measurement periods throughout a given day; therefore, the full diurnal pattern of gaseous emissions was not investigated in the present study. The focus was to replicate conditions which would be followed when using the PAC for gaseous measurements on commercial farms whereby measurements are likely to be conducted between the morning and afternoon (8 h to 16 h).

The CH_4_ output calculated over 50 min in this study was equivalent to 8.62 g/day, which is similar to the amounts calculated by Jonker et al. (2018) and Lockyer (1997) in lambs aged between 6 and 12 months. Alternatively, when animal’s gaseous emissions were measured in respiration chambers their CH_4_ output was found to range from 14 to 24.6 g/day (Pinares-Patiño et al., 2013; Fraser et al., 2015) and 19.0 g/day when measured using the SF_6_ tracer technique (Lassey et al., 1997). According to Doreau et al. (2018) and Goopy et al. (2011) short-term measurements are less accurate at predicting daily CH_4_ production when compared with that of long-term measurements such as respiration chambers due to peaks of emissions during and post feeding which can be missed using spot-sampling methods. Furthermore, short-term measurements add additional sources of variation to the overall daily output as stated by Hegarty (2013). Therefore, the PAC values observed in this study, while comparable to Jonker et al. (2018) and Lockyer (1997) are different to previous studies using respiration chambers and SF_6_ tracer technique, albeit more beneficial for measuring large cohorts of animals across a range of separate farms particularly when obtaining data for genetics based studies. The disparity of the results in the present study and those reported by Fraser et al. (2015), Pinares-Patiño et al. (2013), and Lassey et al. (1997) could be due to a multitude of reasons such as animal age, live-weight, diet type, the breed of animal used, and the measurement technique.

Dry matter intake (DMI) has been shown to account for 76% to 91% of the variation in CH_4_ output in sheep grazing pasture (Muetzel and Clark, 2015; Swainson et al., 2018). In this study, DMI was not measured during the experimental phase; however, in the week prior to the commencement of the experiment, DMI was measured with an average daily intake of 2.91 kg fresh weight and 0.68 kg DM. Methane yield of lambs less than 1-yr old ranged from 21 to 25.8 g/kg DMI (Muetzel and Clark, 2015).This lower DMI is likely to have contributed to the lower CH_4_ emissions seen in this study. Higher CO_2_ production compared with the present study was observed by Jonker et al. (2018) where 623 g/day of CO_2_ was produced, this resulted in more O_2_ being consumed by the animals when in the PAC. Animals in their fasting state should have a RQ of approximately 0.73 (Marston, 1939) or lower (Cock et al., 1967; Kim et al., 2015). Jonker et al. (2018) found a RQ of 0.56 for lambs; however, the RQ in this study was 0.66; which is low considering animals were not fully fasted and were only removed from feed for 1 h prior to measurement. Robinson et al. (2015) showed that taking animals off pasture 1 h before being measured in the PAC caused minimal disruption for the animals and results in more repeatable measurements than overnight fasting. As expected, the total gas production of 14.6 mol/day was similar to that achieved for lambs, however, it was much lower than the 23.3 mol/day observed for ewes (Jonker et al., 2018).

### Relationships between measurement time-points

The relationship between measurement time-points was calculated using the Lin’s concordance correlation (Tables 1–3), Pearson’s correlations using the PROC GLM method were also calculated; however, the correlation coefficient calculated using both these methods were very similar. For example for the correlation calculated, using either the Lin’s or Pearson approach, between methane emissions across time-points differed, on average, by 0.01. However, the correlation coefficients estimated using the Lin’s approach includes a bias correction factor for comparing two methods (Wright et al., 2019) and is, therefore, more applicable in the current study, although comparison of the Lin and Pearson correlation coefficients suggests that the calculated bias in the current study was small. The possibility of reducing the total length of time in which a sheep is placed in the PAC was investigated in this study. Reducing the time a sheep spends in the PAC would reduce any potential stress on the animal but it would also allow a higher throughput of animals through the PAC per day. Goopy et al. (2011) showed that the time in the PAC could be reduced from 2 h to 1 h, the present study investigated the possibility of reducing this time further. Based on the results of this study, for CH_4_ and O_2_ only, it would seem that it is possible to reduce the time from 50 min to 37.5 min without having any negative impact on the data obtained from both gases. This aligns with Robinson et al. (2015) who concluded that there would be a possibility of reducing the time to 40 min when looking at CH_4_. However, CO_2_ also needs to be considered and the present study showed that the intra-day variability of CO_2_ had an impact when animals were only in the PAC for 37.5 min, the correlation between AM and PM measurement runs reduced from 0.75 to 0.55. The production of CO_2_ is linked to energy metabolism (Madsen et al., 2010) but can also be used as an internal marker to estimate CH_4_ production (Madsen et al., 2010; Blaise et al., 2016). A reduction in the time the animal needs to be in the PAC from 50 min to 37.5 min would, therefore, compromise not only the CO_2_ data but would also result in an inability to use the values observed as a proxy for feed intake in future studies. Further reductions of the time to 25 min or 12.5 min would not be possible due to the poor correlations seen for all three gases but in particular for CO_2_. The sensitivity and accuracy of the Eagle 2 monitor also needs to be considered when reducing the time in the PAC. The monitor has an accuracy of ±5%; however, a reduction in the time could potentially compromise the accuracy of the equipment being used. Therefore, results from this study suggest that sheep must remain in the PAC for 50 min to ensure a consistent ranking estimate.

### Variables used to calculate the gaseous output of an animal

Paputungan et al. (2015) showed that a close to unity correlation (0.96 and 0.99) between BV and live-weight in cattle; strong correlations were also calculated in the present study (0.63 to 0.79) albeit slightly lower than those previously reported. Previous studies have assumed that the live-weight of the animal is equal to the volume of the animal (Goopy et al. 2016; Robinson et al., 2016; Jonker et al., 2018). This is not the case in the present study, which seen a large difference between the values for live-weight and the values for BV. Body volume, as measured in this study, only takes into account the thoracic region and does not account for the whole volume of the animal as body parts such as the head and legs are excluded from the calculation; however, this is not the case in the measurement of the live-weight of an animal. From the results observed in this study, live-weight is a better indicator of gas displacement within the PAC. Having to measure animal live-weight is not only quicker but less labor intensive compared with measuring the individual BV for each animal, especially for larger scale studies involving thousands of animals. Live-weight can be used to express CH_4_ emissions per kg metabolic weight, where metabolic weight is equal to live-weight^0.75^ and is represented as CH_4_ g/kg LW^0.75^ (Fitzsimons et al., 2013). Furthermore, live-weight can be used in prediction equations to predict methane output of an animal (Yan et al., 2006; Moares et al., 2013) and to calculate carbon dioxide production (Garnsworthy et al., 2019).

### Intra-day variability of gaseous measurements

The understanding of intra-day variability of gas emissions is extremely important especially when using the PAC as the measurements represent a point in time. Determining the variation observed between morning and evening measurements enables a better understanding of the gaseous output of the animal throughout the day thus eliminating the potential of having to measure animals twice daily in the PAC. The present study showed that the AM and PM measurements for CH_4_ and O_2_ did not differ, with strong correlations between the measurements for both T5-T1 and T4-T1; however, the present study did show a 25% increase in CH_4_ production in the PM measurements compared with the AM (Table 4). This increase in CH_4_ production in the PM measurement corroborate a previous study by Gunter and Beck (2018) who showed a 16% increase in grazing ruminants, while ruminants fed meal-based diets could have an increase of 160% (Hales and Cole, 2017). Lockyer (1997) showed that there was diurnal variation of emissions in grazing sheep with emissions increasing with daylight to reach peak around sunset and declining around sun rise while Manafiazar et al. (2020a) found peak CH_4_ emissions and O_2_ consumption between 0830 and 0900 h. Subsequently, Jonker et al. (2014) stated that *ad libitum* feeding reduced the circadian variation of CH_4_ compared with that of animals with infrequent feeding times. This is likely why a strong correlation is seen between AM and PM measurements in the present study as the animals were on an *ad libitum* diet throughout the experimental period apart from when the feed was removed for 1 h prior to the PAC measurement run. In addition, there was no variation observed between AM and PM measurements for CO_2_. Hegarty (2013) stated that CO_2_ production is less variable than that of CH_4_ as it is related to the animal’s metabolic energy requirements. Corbett et al. (1971) showed that CO_2_ production increased with the activity of the animal while Xu et al. (2017) showed that there were two modest peaks in CO_2_ emissions around feeding time.

### Factors affecting methane production

Upon completion of the data analysis factors which potentially affect CH_4_ production were evaluated. The day in which the measurement took place was shown to affect CH_4_ production in the current study which corroborates with the findings of Goopy et al. (2016) who found that not only the day but the interaction of day and time of measurement had an effect on CH_4_ output. Blaxter and Clapperton (1965) found that the CV for day-to-day variation of CH_4_ for both sheep and cattle was ±7.2%. The time of measurement was shown to impact on CH_4_ production in the current study. Short-term spot measurements can vary depending on the time of day due to the diurnal pattern of CH_4_ production (Hammond et al., 2015); this aligns with results from Goopy et al. (2016) who showed that the time of day impacted on the 1 h measurements in the PAC. Animals had *ad libitum* access to feed in the present study with the exception of when feed was removed for 1 h prior to PAC measurement. Jonker et al. (2014) observed that providing an animal with *ad libitum* access to feed reduced the circadian variation of CH_4_ production and indicated that this method of feeding would be appropriate when using spot sampling methods, albeit using the “Greenfeed” system rather than the PAC technique. The impact of live-weight on CH_4_ production is in agreement with Goopy et al. (2016), while Moorby et al. (2015) found a significant but poor correlation between CH_4_ production and live-weight. As the weight of the animal increases so does voluntary intake (Blaxter et al., 1966), as DMI has been shown to be the main driver of CH_4_ production (Jonker et al., 2018); therefore, CH_4_ production is expected to increase in accordance with the live-weight of the animal. Relative humidity was shown to influence CH_4_ production in the current study (*P* < 0.001). Lockyer and Champion (2001) showed that changes in humidity could alter the amount of CH_4_ produced by sheep. In the present study l/hour values were used that were not standardized to standard temperature and pressure (STP), and may explains the association between humidity and CH_4_ in the present study; however, when g/day values were investigated these values are standardized at STP. As measurements obtained using the PAC technique cannot account for environmental- or weather-associated conditions, the gaseous measurements in g/day are directly affected by the ambient conditions on the day of measurement.

## Conclusion

The current study shows that in order to achieve optimum results from the PAC, sheep must be placed in the chamber for at least 50 min, live-weight should be recorded and used in the calculation of an animal’s gaseous output and the intra-day variability of gaseous measurements does not impact on the ranking of animals for gaseous emissions given the specific feeding and measurement protocol used in this study.

## Abbreviations

BV: body volume
DM: dry matter
DMI: dry matter intake
GHG: greenhouse gas emissions
PAC: portable accumulation chamber
RQ: respiratory quotient
SOP: standard operating procedure
